# A Multi-Sensor Wearable System for the Quantitative Assessment of Parkinson’s Disease

**DOI:** 10.3390/s20216146

**Published:** 2020-10-29

**Authors:** Han Zhang, Chuantao Li, Wei Liu, Jingying Wang, Junhong Zhou, Shouyan Wang

**Affiliations:** 1 Institute of Science and Technology for Brain-Inspired Intelligence, Fudan University, Shanghai 200433, China; 17110850009@fudan.edu.cn (H.Z.); wangjingying@fudan.edu.cn (J.W.); 2 Naval Medical Center of PLA, Department of Aviation Medicine, Naval Military Medical University, Shanghai 200433, China; chuantaoli@hotmail.com; 3 Academy for Engineering and Technology, Fudan University, Shanghai 200433, China; 18110860047@fudan.edu.cn; 4 Hebrew SeniorLife Hinda and Arthur Marcus Institute for Aging Research, Harvard Medical School, Boston, MA 02131, USA; junhongzhou@hsl.harvard.edu

**Keywords:** multimodal sensors, Parkinson’s disease, telemedicine, wearable sensors

## Abstract

The quantitative characterization of movement disorders and their related neurophysiological signals is important for the management of Parkinson’s disease (PD). The aim of this study is to develop a novel wearable system enabling the simultaneous measurement of both motion and other neurophysiological signals in PD patients. We designed a wearable system that consists of five motion sensors and three electrophysiology sensors to measure the motion signals of the body, electroencephalogram, electrocardiogram, and electromyography, respectively. The data captured by the sensors are transferred wirelessly in real time, and the outcomes are analyzed and uploaded to the cloud-based server automatically. We completed pilot studies to (1) test its validity by comparing outcomes to the commercialized systems, and (2) evaluate the deep brain stimulation (DBS) treatment effects in seven PD patients. Our results showed: (1) the motion and neurophysiological signals measured by this wearable system were strongly correlated with those measured by the commercialized systems (*r* > 0.94, *p* < 0.001); and (2) by completing the clinical supination and pronation frequency test, the frequency of motion as measured by this system increased when DBS was turned on. The results demonstrated that this multi-sensor wearable system can be utilized to quantitatively characterize and monitor motion and neurophysiological PD.

## 1. Introduction

Parkinson’s disease (PD) is the second most common type of neurodegenerative disorder [1], and its prevalence increases with advancing age [2]. PD severely impairs motor control function, leading to multiple movement disorder symptoms, including tremor, freezing of gait, and bradykinesia. These disorders often diminish the quality of life and increase the risk of mortality and morbidity in patients with PD. It is thus of great significance to characterize these symptoms, which will ultimately help in the clinical diagnosis and management of this disease [3,4].

Clinical rating scales, such as The Movement Disorders Society Unified Parkinson’s Disease Rating Scale (MDS-UPDRS) [5], a gold standard for PD characterization, have often been used to measure the severity of movement disorders in PD in clinics. However, these types of scales have limitations. These scales are not that sensitive to the subtle changes in motor function in the early stages of PD; these assessments are often conducted in laboratory or clinical settings; and the interpretation of the results relies on the experience of rating personnel [6,7,8,9]. Novel strategies using sophisticated portable devices to objectively quantify movement disorders in PD are therefore in high demand [10,11,12,13].

Recently, wearable sensor systems consisting of inertial measurement units (IMUs) measuring the acceleration and angular velocity of motion have been used to characterize motor function in patients with PD. A longer-term monitoring time of symptoms can be achieved, and subtle changes in the progression of PD can be detected [14,15,16,17,18,19,20,21,22,23,24,25,26]. The Parkinson’s KinetiGraph™, for example, uses a small sensor system attached to the wrist to assess the status of motor fluctuations, tremor, and dyskinesia in PD patients for long-term monitoring [27]. Using multiple IMU sensors to form a “body network area” enables objective assessment of the progression of PD over the long term [28]. On-shoe IMU sensors provide rich information about gait, resulting in more insight into other features of PD [29].

Meanwhile, multiple neurophysiological signals, including those from musculoskeletal (e.g., electromyography, EMG), cardiovascular (e.g., electrocardiogram, EKG), and cerebral systems (e.g., electroencephalogram, EEG), are also important for the characterization of PD [30,31,32,33,34,35]. Muscle activity as captured by EMG, for example, has been linked to tremor motion, rigidity, and balance in patients with PD and can be used to identify the subtypes of PD [36,37]. Therefore, it will be of great significance to develop a novel sensor system with the functionality to measure signals related to both movement and neurophysiology in PD.

The primary concern about sensor-based devices is their usability to monitor multiple parameters in the clinical environment and their ability to track the course of PD over the long term [13,38]. Therefore, the main problems of current wearable devices for PD symptom measurement are that commercially available devices used to monitor PD have few monitoring parameters and cannot detect some of the symptom changes that result from disease progression, such as asymmetric symptoms [13]. Current multiparametric device detection solutions mostly use general-purpose motion sensors, sometimes paired with other systems to monitor electrophysiological signals; however, there is a lack of wearable devices that can achieve high-precision motion and electrophysiological integration. The problem with generic multiparameter wearable devices is that the calculated parameters are some common parameters which cannot greatly cover the usual PD test items, which makes the assessment of PD symptoms difficult. More importantly, the information collected by these devices is stored locally, which is not ideal for multicenter experiments and big data analysis.

We thus developed a novel wearable sensor system consisting of five motion sensors and three high-resolution electrophysiology sensors with the functionality of wireless data transmission. This novel system enables the long-term simultaneous monitoring of motion (e.g., hand motion, gait speed, body sway) and neurophysiological data (e.g., EEG, EKG, EMG) in people with PD. The data captured by this system can be automatically uploaded to a cloud-based system via Wi-Fi for analysis and to obtain the outcomes pertaining to the clinical and functional characteristics of PD. Here, we introduce the development and functionality of each hardware component and the results of preliminary studies designed to examine the validity and reliability of this novel system.

## 2. System Development

### 2.1. System Overview

**Figure 1:**
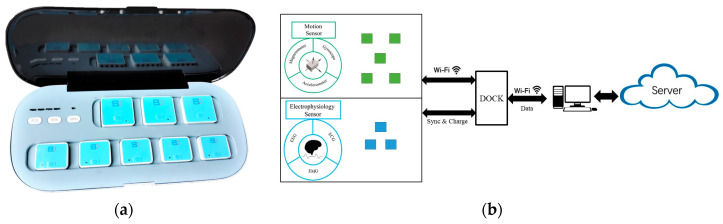
(**a**) The hardware including the sensors and dock. (**b**) Overview of the system diagram.

Figure 1 shows the infrastructure and workflow of this system. Multimodal signals can be simultaneously captured by wearable sensors and transferred via Wi-Fi. Then, the cloud-based server and software platform can complete the analysis and management of the data and generate reports containing the important outcomes.

### 2.2. Hardware Framework

We developed two types of sensors to capture motion signals and electrophysiology signals. Each sensor is connected to the PC through wireless technology. The sensors can be used separately or together; therefore, users can perform various functional tests with the use of different sets of sensors. During the test, the data are transferred in real time with very low time lags.

#### 2.2.1. Motion Sensors

**Figure 2:**
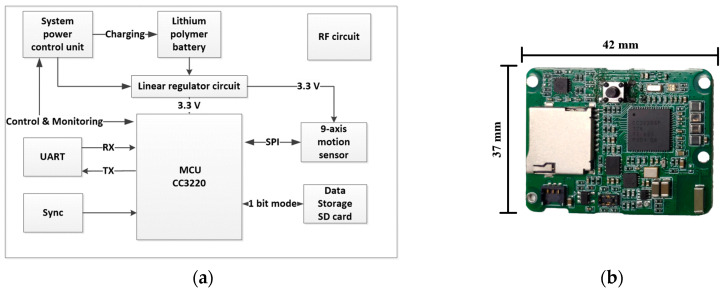
(**a**) Hardware diagram of the motion sensor. (**b**) The motion sensor.

The motion sensor units consist of five independent sensors and are 45.01 × 36.30 × 15.09 mm in size with a weight of 27.8 g. Figure 2a shows a diagram of the hardware framework. The microcontroller unit is the CC3220 MCU (microcontroller unit) (chip CC3220, Texas Instruments, Dallas, TX, USA). The system is powered by a chargeable 600 mA Li-ion battery. The system power control unit is managed by the MCU and docking station, and the unit regulates battery charging and monitors battery voltage. The sensor has two timer systems. One is a real-time clock (RTC), providing a low-precision and low-power consumption clock. The system uses RTC to add a time label to each test. The other is a high-precision timer. It is set by a synchronization signal issued by the docking station. The sensor combines two timer systems to guarantee that different sensors can record data with a low time difference. In addition, the sensor uses a Secure Digital (SD) card to store all sampled data, so the sensor can record data without sending them through Wi-Fi. This mode will support a long-term continuous activity record, and the motion sensors can continue recording for 22 h. If wireless data transmission is interfered with by the environment, data transfer will undergo severe data loss, and the data on the SD card will protect the test results.

The motion sensor uses the MPU9250 chip (chip MPU-9250, IvenSense Inc, San Jose, CA, USA) to acquire motion information; it houses three 3-axis sensors. The 3-axis gyroscope to measure three-dimensional dynamic angular speed can describe twisting or rotational movement; the 3-axis accelerometer can be used to reconstruct sensor space motion statuses; and the 3-axis magnetometer can detect fluctuations in the Earth’s magnetic field. The magnetometer data are often fused with the gyroscope and accelerometer data to deliver absolute heading and navigation. Nine high-resolution analog-to-digital converter (ADC) digitize the accelerometer, gyroscope, and magnetometer outputs. The maximum full-scale values are ±16 g, ±2000 dps (degrees per second) and ±4800 μT, respectively.

#### 2.2.2. Electrophysiology Sensors

**Figure 3:**
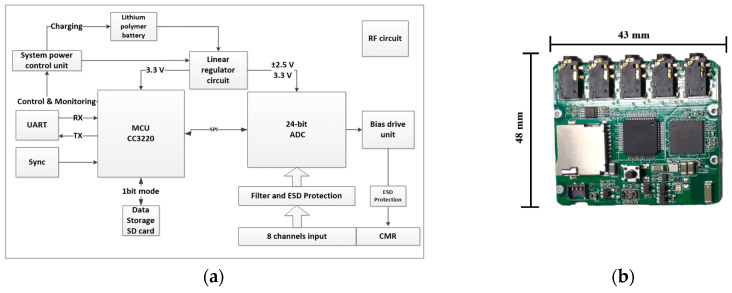
(**a**) Hardware diagram of the electrophysiology sensor. (**b**) The electrophysiology sensor.

The electrophysiology sensor unit includes three individual sensors. The dimensions of the sensor are 51.08 × 44.79 × 15.09 mm, and the weight is 41.2 g. The diagram of the hardware architecture is illustrated in Figure 3. The main component of the analog-to-digital converter circuit is the ultralow input-referred noise (1μVPP), high-resolution (24-bit), low-power consumption ADS1299 chip (chip ADS1299, Texas Instruments, Dallas, TX, USA). The sensor benefits from the use of this integrated chip by simplifying the front-end circuit and reducing the size of the sensor. Each sensor supports eight channels of bipolar or monopolar input and programmable input amplifiers. The sensor can acquire common-mode signals from sampled signals and provide feedback to the body by a negative feedback amplifier circuit to suppress the common-mode noise. The sensor contains a one-order lowpass filter that removes the signal we are not focusing on. The sensor can vary the sampling rate from 250 to 2000 Hz, and the system can realize 24 channels of raw data transmission through Wi-Fi simultaneously in real time. The design of the hardware RF circuit and MCU-related circuit is the same as that of the motion sensor. The power management unit provides 3.3 V for the digital circuit and high-precision ±2.5 V low-dropout regulator (LDO) for the ADC analog reference. In long-term continuous activity record mode, the electrophysiology sensors can continue recording for 25 h.

#### 2.2.3. Docking Station

The dimensions of the dock are 36 × 16 × 7 cm, and the weight is 2.5 kg. The dock is powered by a battery, and it is capable of 22 h of continuous operation in real-time system mode. The docking station function is to manage sensors and connect the sensors to the computer. The sensors communicate and transmit the data via the Wi-Fi router to the computer client. The socket on the docking station can charge the sensor through the pin connector. Moreover, it can send a trigger signal to sync all sensors with a high-precision timer. This function can ensure a sensor time difference of less than 1 ms in every test.

### 2.3. Firmware

To achieve concurrent transmission of high-volume data from five motion sensors and three 8-channel high-resolution electrophysiology sensors, we used Wi-Fi to realize high-throughput wireless data transmission.

A CC3220 MCU (chip CC3220, Texas Instruments, Dallas, TX, USA) was used in each sensor. CC3220 contains two separate execution environments: a cortex^®^-m4 MCU and a CC3120 network processor. The MCU had 100 DMIPS computing speed for signal acquisition, real-time processing, and dynamic interaction with computer software. The network processor can reach 13 Mbps under the TCP/IP protocol, which can realize real-time data transmission.

**Figure 4:**
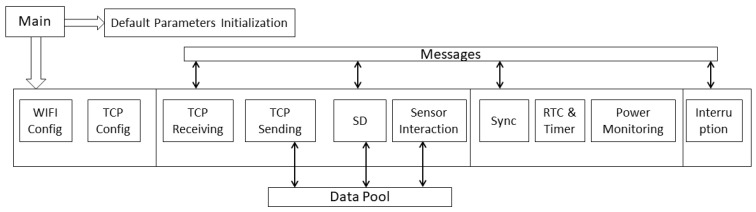
Diagram of the firmware in the CC3220.

To guarantee a stable sampling frequency, especially for the electrophysiology sensor, and transmit the data, a real-time operating system (FreeRTOS) was adopted. Therefore, the data acquisition will not be blocked by time-consuming processes such as interacting with low-speed peripherals and wireless connecting processes. The different functions are assigned to the different tasks shown in Figure 4. The cortex kernel will dispatch the task according to predefined priorities and task running state. In the software system, the data acquisition process has the highest priority, followed by the data storing process and the wireless transmission process. The high-precision clock must sync before each test starts, and the computer client can set the RTC, so the data acquisition in independent sensors will not experience a time difference. The wireless connection was based on the TCP/IP protocol stack that runs in the network processor and does not consume the primary processor computing resources.

## 3. Experimental Protocol

We completed three preliminary tests to examine the capacity of this novel wearable sensor system for simultaneous data acquisition, the validity and reliability of the data recorded by the sensors, and the feasibility of using this system to assess motor symptoms and the effects of deep brain stimulation on these symptoms in PD patients in clinical settings.

### 3.1. Experiment 1: Synchronous Acquisition Test

**Figure 5:**
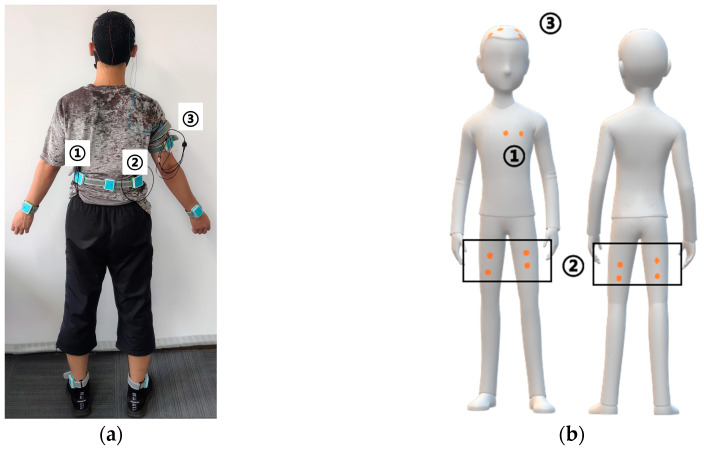
(**a**) Placement of the eight sensors; the numbers identify the three electrophysiological sensors. (**b**) Diagram of the electrode placement position of the electrophysiological sensors.

One healthy younger adult (age: 21; sex: male) without any neurological diseases completed this test. As shown in Figure 5, five motion sensors were fixed on the left wrist, right wrist, left ankle, right ankle, and waist. Channel 1 of the first electrophysiology sensor was used to measure 2-lead ECG signals, and channel 1 to channel 4 of the second electrophysiology sensor were used to monitor the EMG of the quadriceps femoris muscle and biceps femoris muscle on both legs. Channel 1 and channel 2 of the third electrophysiology sensor were used to measure the bipolar EEG signal, which was placed at the C2, C4, C1 and C3 positions of the 10/20 EEG template. During the test, the participant first sat quietly for 10 s and then stood up and completed a 10-m timed up and go test. The signal from sitting to standing, straight walking, turning, and standing to sitting was measured by the system.

### 3.2. Experiment 2: Motion Sensor Validation Test

In this experiment, one participant completed five trials of the 10 m walking test. The reference system for the motion sensor was an optical motion capture system (Qualisys Mocap, Qualisys, Göteborg, SE), and the optical motion capture system is the gold standard in kinematics evaluation. The motion sensor was evaluated during 10 m walking tests [39] and was recording from a standing start to a standing finish and included gait initiation and termination. This experiment was conducted in a motion lab where a mounted Qualisys Mocap motion capture system was used. Nine cameras tracked the marker trajectories at a frame rate of 100 Hz/s. The five motion sensors were fixed on the left and right wrists and ankles and the fifth sensor was placed over the fourth lumbar vertebrae. The reason for choosing these locations was because they are relatively close to the bone; the sensor could record the movement of the bone more precisely than many other regions. The motion sensor sample rate was configured to 100 Hz, the same as the motion capture system. The passive markers were placed on specific anatomic points on the subject’s body and each sensor surface center. The participants performed the 10 m walking tests five times, while both systems acquired kinematic data. In each trial, the participant walked alongside the global coordinate system of the optical motion capture system to compare the measurement results of the two systems.

### 3.3. Experiment 3: Electrophysiology Sensor Validation Test

For the electrophysiology sensor, we conducted two different tests. First, we used a high-performance signal generator (EasyCap SIGGI-II, EASYCAP GmbH, Woerthsee-Etterschlag, DE) to generate different frequencies from 1 to 400 Hz sinusoid waves with 100 μV vibration amplitude for the generated signal test. Second, we compared our sensor with the state-of-the-art physiology measurement system (BIOPAC MP160, BIOPAC Systems, Inc., Goleta, CA, USA) to evaluate the correlation between the signals. We evaluated our sensor during a wrist extension and clenched fist test. For this test, two participants completed 10 trials. For wrist extension testing, we used one channel of an electrophysiology sensor and placed the differential input electrodes at the extensor of the right forearm. The specially designed electrode lead connected the electrodes to our sensor and BIOPAC. The sensor and BIOPAC sample rate was set to 2 kHz for data alignment. Two subjects participated in this experiment. In each trial, the subject was asked to sit steady, put their right forearm on the table, perform a wrist extension, and then clench their fist for 30 s.

### 3.4. Experiment 4: Clinical Application

After assessing the accuracy of each sensor and parameter, a more systematic test on the motion sensors was conducted in Nanjing Brain Hospital under the supervision of neurologists; all subjects provided informed consent, and the result was uploaded and managed on the cloud server. During this real-world experiment, we focused on the system feasibility for assessing PD symptoms in the clinic. The aim was to show that the system was capable of quantifying PD symptoms. In this test, we selected supination and pronation frequency as a measure of bradykinesia to assess the deep brain stimulation (DBS) effect [40]. Seven individuals (four females, three males) aged between 51 and 68 participated in this test. The patients were not treated with dopaminergic therapy or tested in ON/OFF condition. The stimulation parameters are different for each subject and are optimized by the physician according to the specific conditions of the patient. Each patient underwent DBS with bilateral quadripolar electrode placement in the STN. The motion sensors were affixed to two wrists, supervised by neurologists, and then the patient performed supination and pronation following task 3.6 of the MDS-UPDRS, while stimulation was ON and OFF.

### 3.5. Data Processing

To compare different measurement systems in the motion sensor test, we selected a set of marker clusters around each sensor to form rigid bodies that were used to measure the angular velocity and acceleration of body parts wearing the motion sensor in the global coordinate system. In every trial, the subject was asked to walk alongside the global coordinate axis. Thus, the sensor measurement results were matched with the motion capture system outputs. The marker trajectory data were smoothed by a Savitzky–Golay smoothing filter to remove noise [41]. The time of gait initiation was manually selected, and the first peak was used for alignment. For the electrophysiology sensor test, we simply applied a one-degree Chebyshev Type I filter with 1 and 5 kHz for the bandpass, which was then followed by a 50 Hz notch filter.

**Figure 6:**
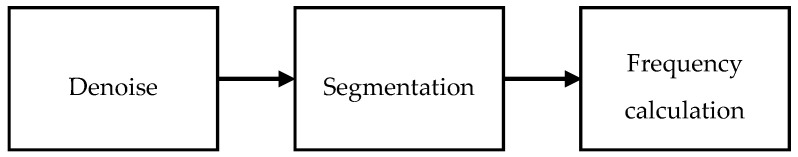
The procedure for data processing.

In the real-world experimental test, we used the supination and pronation frequency as the DBS treatment evaluation parameter. Figure 6 shows the procedure for data processing. First, we removed the noise from the signal of the major axis in rotation movement by bandpass filtering, and the passband frequency was between 0.1 and 12 Hz. The next step was rotation detection. We used a threshold to count the total frequency of supination or pronation, and then we obtained the rotations *n* by doubling the total number of supination or pronation counts. The threshold was the mean of this signal plus standard deviation (SD) [42]. Finally, the frequency was calculated by dividing *n* by the time duration.

#### Statistical Analysis

In the sensor comparison test, we computed the Pearson correlation between the sampled data from our sensor and the data acquired from the commercial equipment. This method was used to determine whether our sensor can acquire excellent quality signals.

In the DBS treatment experiment, we used the angular amplitude and symmetry index (1) to compute the asymmetric conditions before and after DBS was ON.

The symmetry index [43] was calculated as follows:

(1) $$SI_{index}=100\times \frac{\left|X_{L}-X_{R}\right|}{\max\left(X_{L},\;X_{R}\right)}$$

where $X_{L}$ represents the mean frequency on the left side, and $X_{R}$ represents the mean frequency on the right side.

## 4. Results

**Figure 7:**
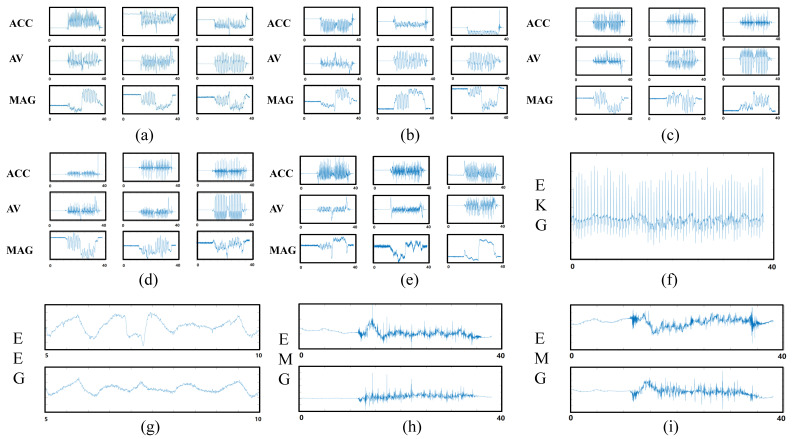
Example of signals measured by the sensors in this system. (**a**) Left wrist motion sensor. (**b**) Right wrist motion sensor. (**c**) Left ankle motion sensor (**d**) Right ankle motion sensor. (**e**) Trunk motion sensor. (**f**) Electrophysiology sensor 2-lead ECG. (**g**) Electrophysiology sensor bipolar input on C2, C4, C1, and C3 positions on the 10/20 EEG template. (**h**) Electrophysiology sensor bipolar EMG input from the left leg. (**i**) Electrophysiology sensor bipolar EMG input from the right leg.

The system enabled successful simultaneous data capture. Figure 7 shows one set of example data captured by the sensors. Different sensors were capable of recording at the same standardized time, and the events recorded by each sensor were temporally aligned.

**Figure 8:**
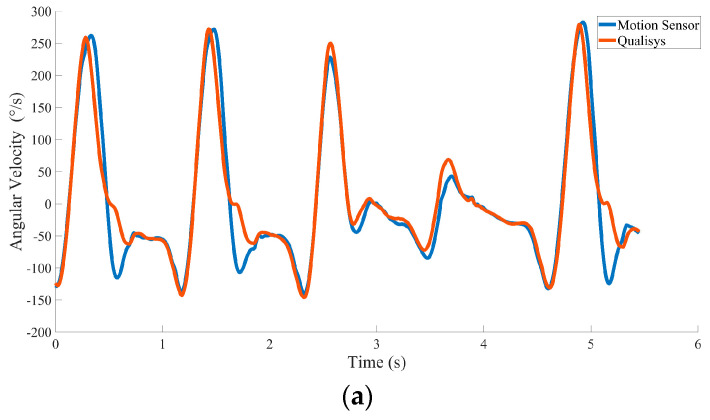

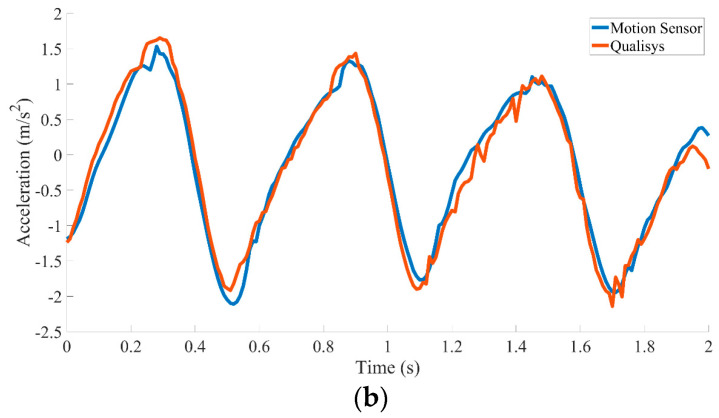
Data from a representative participant showing a comparison of the angular velocity and acceleration between the motion sensor and optical motion capture system. The blue line represents the motion sensor, and the orange line represents the optical motion capture system. (**a**) The angular velocity between the right leg and transverse plane in the 10-m walking test. (**b**) The acceleration of walking direction separated from the waist test point.

Figure 8 shows one example of the acceleration and angular velocity signals depicting one trial of motion on the dominant axis during the 10-m walk test. The aligned angular velocity measured by the motion sensor (peak = 224.57 ± 1 52°/s; trough = −130.84 ± 39.75°/s) and optical motion capture system (peak = 224.68 ± 161°/s; trough = −122.12 ± 49.33°/s) was similar. Peaks and troughs showed highly significant correlations (the mean correlation was 0.92). The acceleration comparison results showed that the peaks (motion sensor = 1.07 ± 0.67 m/s^2^; optical motion capture system = 1.23 ± 1.2 m/s^2^) and troughs (motion sensor = −1.86 ± 0.36 m/s^2^; optical motion capture system = −1.76 ± 0.34 m/s^2^) did not show a large difference either, with a highly significant correlation (the mean correlation is 0.92, *p* ≤ 0.001).

In five 10 m walking tests, we selected five to seven seconds of data to make the comparison. The data length we chose was related to the walking speed during the test and excluded the data while the body was rotating. Table 1 shows the correlation coefficients of the aligned data between the motion sensor and the optical motion capture system. Table 1 shows that the measurement results collected by the two systems were significantly correlated (*p* < 0.001). Table 2 shows the average of the absolute value of the difference between the data recorded by the two systems, and the standard deviations of acceleration and angular velocity were 0.063 and 3.9092, respectively. The difference between the data measured by the two systems is small. Therefore, our sensor stably acquired accurate motion data across multiple tests.

**Figure 9:**
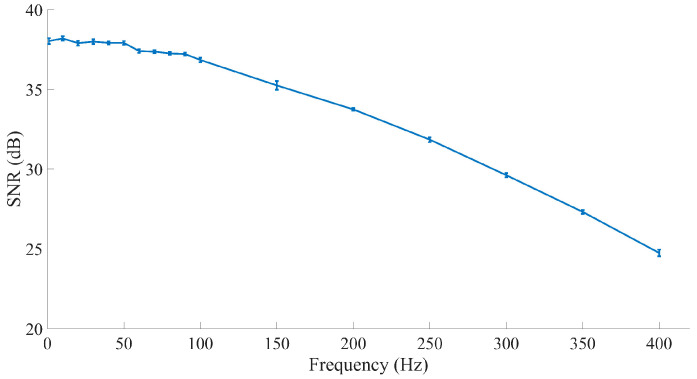
The SNR of the generated signal. The electrophysiology sensors for the EEG signals contained a first-order lowpass filter that removed high-frequency signals that we do not focus on in EEG. The blue line represents the SNR of each acquired signal across frequencies.

The generated signal was measured by the electrophysiology sensor, which contained a first-order 300-Hz lowpass filter, then digitized and transmitted to the computer client through Wi-Fi. Figure 9 shows the generated signal that the sensor obtained. The results showed that the frequency properties of the generated signals obtained by our sensor were accurate for restoring the original signal with a fine signal-to-noise ratio (SNR).

**Figure 10:**
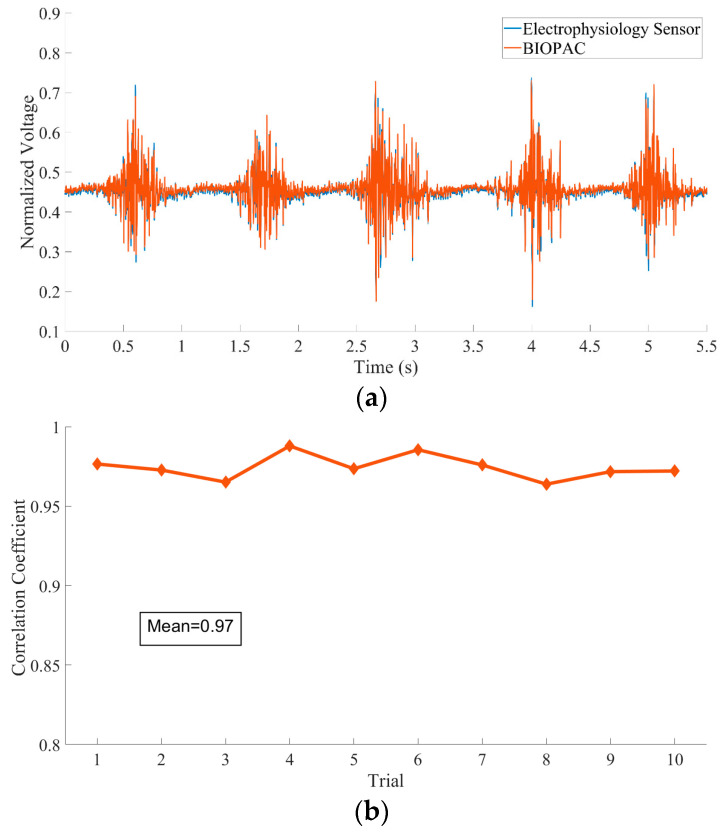
(**a**) A comparison of 11-s EMG signals acquired by the electrophysiology sensor and BIOPAC EMG module. (**b**) Correlation coefficient between the EMG recorded from the two sensors. Recordings from the two sensors show a remarkably high correlation.

In the comparison test, Figure 10a plots 11 s of EEG data acquired by the two systems where there is a high correlation between the two signals. Figure 10b plots the correlation across all trials. The correlations between the surface EMG signals acquired by the two different systems were very high (the mean correlation reached 0.97, *p* < 0.001), indicating that the sensor developed in this research is capable of measuring EMG signals from the surface skin.

### System Application in the Clinic

**Figure 11:**
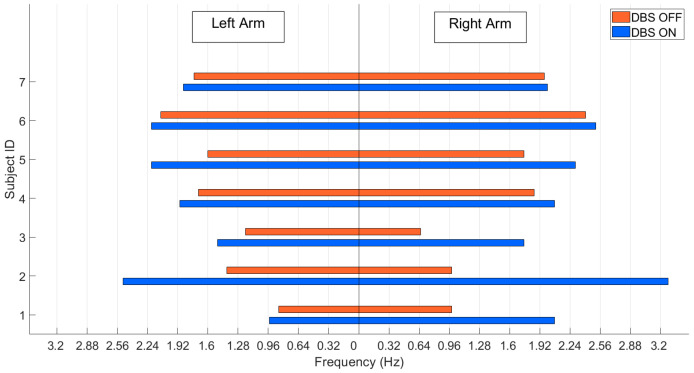
Comparison of supination and pronation frequency.

The UPDRS-III score significantly decreased after the DBS was ON compared to the score before the DBS treatments (t15 = −15.22, *p* < 0.001). As shown in Figure 11, while DBS was ON, the frequency of the patient’s hand rotation increased. The amplitude differentially changed across sides, indicating the asymmetrical symptoms of the disease. The mean value of the symmetry index during DBS OFF was 38.74, and the standard deviation was 21.79. When DBS was ON, the mean value of the symmetry index decreased to 19.86, and the standard deviation was 18.97. Based on the change in the symmetry index, we observed that the asymmetrical condition of the patients improved when DBS was ON. The test results provided evidence that the proposed system can provide quantitative outcomes for PD patient symptom analysis. The system calculated parameters applicable to the classic PD symptom exam, and the system acquired parameters that are difficult for people to observe.

## 5. Discussion

We developed a novel wearable sensor system to measure the motion and neurophysiological signals in people with PD, aiming to characterize the function and disease severity of PD, and address the deficiencies in current PD symptom measurement devices, such as the few measurement parameters and the difficulties in transmitting and managing measurement results. Our device was designed to solve these problems as well as to hopefully accelerate PD research. Our system achieved these goals with the following approaches.

Multiple sensors were developed for this system, allowing a more complete characterization of people with PD. In the motion sensors, an accelerometer, gyroscope, and magnetometer were used together to measure twisting motions with low drifting errors. Through those motion sensors, we obtained reliable parameters such as acceleration, rotational movement, and twisting. Electrophysiology sensors can assess the electrical activity of different muscle groups, which can be used to study abnormal muscular tone to help physicians diagnose PD with atypical Parkinsonism and other trembling conditions [13,34,44]. In addition, our sensors can monitor nonmotor symptoms. High-resolution wearable electrophysiology sensors can monitor multichannel ECG or EEG signals. The sensors could be a useful tool for PD nonmotor symptom (e.g., sleep problems or anxiety) research [38]. Meanwhile, the distributed sensors in our system can monitor different parts of the body so that we can observe the asymmetry in PD symptoms, which can help assess the progression of the PD course.

Moreover, the compact and wireless design of our sensor provides minimal restriction on testing requirements that allow the patient to perform a test in the area outside of the hospital. More importantly, the outcome is easy to understand by the patient and caregiver. This helps improve self-awareness and make daily measurement easier [28]. Finally, the sensor adopted Wi-Fi as a data transfer method, which makes our sensor a promising tool for real-time closed-loop DBS. Wi-Fi is a universal communication technology that allows our sensor to easily connect to other systems. Furthermore, with Wi-Fi’s fast data transfer speed, a sensor-acquired low-lag signal can be used in a real-time algorithm to achieve more accurate dynamic stimulation.

We have now implemented the functionality of the hardware and performed initial validation testing. However, the sample size was small, and in the next step, we will incorporate cloud storage, recruit more subjects, and use more experimental paradigms for further reliability and reproducibility testing in large populations.

### Study Limitations and Future Work

In this study, we have verified the feasibility of our sensor system in the clinic. Unfortunately, the experiment lacks validation of the clinical use of electrophysiological sensors. Validation of our sensor system in more subjects and a home setting scenario requires further investigations.

Our future work is to develop a standardized protocol to assess the feasibility of the clinical application of our sensors in varied scenarios.

## 6. Conclusions

This study presented a wearable device for Parkinson’s disease symptom measurement that can record multimodal motion signals and high-resolution electrophysiological signals. The sensor components involved wearable technology, and each sensor was compact and lightweight, resulting in less burden for the patient while performing the test. In combination with Wi-Fi technology, the testing can be efficiently conducted in most areas without restrictions, and the sensors can be flexibly matched for different measurement paradigms. Furthermore, high-speed wireless transmission allows us to monitor the multimodal signal in real time. The measurement parameters and analysis results can be uploaded to the cloud server for remote access.

In conclusion, our proposed system aims to provide a useful tool for PD cardinal motion symptom measurement (with five 9-axis motion sensors). Moreover, the system can collect important physiological information (with three 8-channel high-resolution electrophysiology sensors), which provide rich information on nonmotor behaviors. Furthermore, cloud storage offers a reliable method for long-term disease course management and multicenter research.

## Figures and Tables

**Table 1:** The correlation s coefficient from 5 trials.

Quantity	1	2	3	4	5
Acceleration	0.98 **	0.96 **	0.96 **	0.96 **	0.95 **
Angular Velocity	0.96 **	0.94 **	0.93 **	0.95 **	0.95 **

** *p* ≤ 0.001.

**Table 2:** The difference between the two methods from 5 trials.

Quantity	1	2	3	4	5	SD
Acceleration	0.1579	0.1603	0.2402	0.2926	0.2736	0.0630
Angular Velocity	18.07	25.02	27.74	22.87	19.72	3.9092
